# Expansion Improved the Physical and Chemical Properties and In Vitro Rumen Digestibility of Buckwheat Straw

**DOI:** 10.3390/ani14010029

**Published:** 2023-12-21

**Authors:** Xiaohui Cao, Sasa Zuo, Yanli Lin, Rui Cai, Fuyu Yang, Xuekai Wang, Chuncheng Xu

**Affiliations:** 1 College of Engineering, China Agricultural University, Beijing 100083, China; s20193071205@cau.edu.cn (X.C.); zss@cau.edu.cn (S.Z.); 2 College of Grassland Science and Technology, China Agricultural University, Beijing 100093, China; lyl161@126.com (Y.L.); yfuyu@cau.edu.cn (F.Y.); 3 School of Environmental Science & Engineering, Huazhong University of Science and Technology, Wuhan 430074, China; chenmei@cau.edu.cn

**Keywords:** buckwheat straw, expansion, structure, physio-chemical properties, in vitro rumen digestion

## Abstract

**Simple Summary:**

Straw has poor palatability, a high degree of lignification, and difficulty in compression, which limits its feed application. Expansion technology can make the hard straw soft and improve the physical and chemical properties of the straw. At present, there is no comprehensive characterization and evaluation of the physical and chemical properties and feed utilization value of expanded straw, and there is a lack of research on the texture properties of straw itself. The texture properties have important effects on the compressibility of straw during silage, the palatability during feeding, and even the energy conversion efficiency during digestion, which have important guiding significance for the further utilization of straw. We combined mechanical and chemical methods to investigate the hardness, elasticity, chewiness, compressive properties, and structural changes of expanded straw and verified the effect of the changes in mechanical properties and chemical structure of expanded straw on improving digestibility by in vitro digestion. This result is of great significance for the further effective application of expanded straw as a feed resource.

**Abstract:**

The hard texture and poor palatability of straw are important factors that hinder its application in feed. Expansion is a technology that can improve the utilization of biomass, but few studies have comprehensively revealed how to change physicochemical characteristics to improve nutritional value. In this study, mechanical and chemical methods were combined to study the texture properties, rheological properties, and physicochemical structures of straw, and its utilization value was evaluated by in vitro rumen digestion. Expansion caused hemicellulose degradation, cellulose separation, and lignin redistribution, resulting in a decrease in crystallinity. The hardness and chewiness of expanded straw were reduced by 55% to 66%, significantly improving palatability. The compressive stress could be reduced by 54–73%, and the relaxation elasticity was reduced by 5% when expanded straw was compressed. The compression deformation of expanded straw was doubled compared to feedstock, and the compacting degree was improved. Expanded straw significantly improved digestibility and gas production efficiency, which was due to the pore structure increasing the attachment of rumen microorganisms; besides that, the reduction of the internal structural force of the straw reduced energy consumption during digestion. The lignin content decreased by 10%, the hardness decreased further in secondary expansion, but the digestibility did not improve significantly.

## 1. Introduction

Buckwheat (*Fagopyrum esculentum*) belongs to the Polygonaceae family and is a nutritious coarse grain. Numerous studies have shown that buckwheat is rich in effective natural antioxidants and has gradually become a popular source of human food. However, buckwheat will produce a large amount of straw every year after a series of processing steps, and buckwheat straw has become a promising by-product that can no longer be ignored. The rational utilization of buckwheat straw for feed purposes could help reduce feed shortages in livestock production [1]. However, most cellulose is associated with lignin in the form of lignocellulose, restricting the enzymatic conversion due to the unique resistance structure, so removing lignin or increasing the accessibility of holocelluloses to microorganisms can enhance the nutritional value of straw [2,3]. Therefore, how to improve the physicochemical structure of buckwheat straw while retaining the nutrients is particularly important to improving its utilization value as a feed source.

Expansion is a kind of thermo-mechanical pretreatment that relies on the friction between the screw, straw, and inner wall of the machine to provide additional heat and energy, forming a high-temperature and high-pressure environment [4,5]. When the pressure is released instantaneously at the discharge port, the straw explodes, causing the depolymerization of macromolecules and enhancing their biodegradability [6]. The substrate simultaneously undergoes high shearing forces because of the protrusions of the screw during transport, and the twisting action of the screw is conducive to the full mixing of straw and water, thus leading to a better effect upon the explosion. This technique avoids the severe treatment conditions that produce a large number of by-products from steam explosions and liquid ammonia, as well as the limitations of grinding. Expansion is usually used for snack food, feed, and plastic products [7]. It could improve the expansion rate and porosity of oat bran, and the feasibility of oil extraction from oat bran was realized [8]. Regarding application in crop straw, some studies have shown that expansion pretreating corn stover or rice straw can significantly improve the sugar recovery rate and increase biogas production from grass [4,9]. The expansion process interferes with the biomass structure through the combination of thermal energy and mechanical energy, causing a series of physical and chemical changes in the material. However, few studies have comprehensively revealed how to change physicochemical characteristics in the assessment of its nutritional value as a feed source.

The change in mechanical properties of lignocellulosic biomass is closely related to its chemical composition and structure. The lignocellulosic biomass structure is composed of a highly complex matrix of cellulose, hemicellulose, and lignin. These components are interlinked by ether, ester, carbon-carbon, and hydrogen bonds [10]. Therefore, the relationship between stress and deformation of the samples can reflect the binding properties of the lignocellulosic biomass [11]. Generally, traditional chemical methods are usually used to evaluate the effect of pretreatment on the in vitro digestibility of straw feed, and there is a lack of evaluation of mechanical properties for biomass. However, Viamajala et al. [12] showed that the enzymatic hydrolysis system showed shear dilution characteristics through the relationship between rheological properties and initial solid concentration, pretreatment degree, and particle size in the enzymatic hydrolysis system of dilute acid pretreated corn straw. Liu and Chen [13] reported the heterogeneity and mechanical properties of corn stovers and their correlations with the efficiency of steam explosion and hydrolysis. Therefore, changes in mechanical properties may affect the conversion efficiency after the feed enters the rumen, which has important research significance for the improvement of the biological transformation process of the straw nutritional value. Besides, changes in mechanical properties can be used to improve the palatability and the degree of compaction during ensilage.

In this paper, the correlation between chemical composition and mechanical properties was investigated from the perspective of biomechanics, and the internal mechanism of the effect of expansion modification on physical and chemical properties was explored. Furthermore, the effects of cell wall structure and biomechanics on ruminal digestibility were explained, and a new idea was opened up for the evaluation of crop straw nutrition and utilization value.

## 2. Materials and Methods

### 2.1. Buckwheat Straw Preparation

The buckwheat straw was supplied by Fuxin City, Liaoning Province. The stubble height was about 20 cm when harvested and stored in a cool and dry place. Then, the buckwheat straw raw material was processed into a length of 5 cm with a cutter machine.

### 2.2. Different Degrees of Expansion Treatment

The cut buckwheat straw was expanded on the straw expander (Model 9P-150, Liaoning Xianghe agricultural animal husbandry Industry Co., Fuxin, China). The screw diameter of the expander is 150 mm, the spindle speed is 510 r/min, and the output is 1–1.5 t/h. The dried buckwheat straw was evenly put into the feeding port and conveyed by the screw auger to the expanding chamber. Through the extrusion, shearing, and friction of the screw and the screw sleeve, the pressure and temperature in the expanding chamber were continuously increased, and finally, the straw was instantly sprayed from the outlet (EO). The buckwheat straw of the EO was put into the expansion chamber again for secondary expansion (ES). Water was appropriately added to the expansion chamber through the nozzle during both processes to prevent carbonization. The expanding process lasted about 6 s, the temperature could reach above 120~150 °C, and the pressure was higher than 3.4 MPa. Retain part of the original buckwheat straw of three treatment groups in ziplock bags for mechanical analysis, and dry the rest at 65 °C for 48 h.

### 2.3. Chemical Composition

Dried samples were milled to pass through a 1-mm screen. The contents of neutral detergent fiber (NDF) and acid detergent fiber (ADF) were determined according to the improved Van Soet’s methods [14,15], with the addition of alpha amylase but no sodium sulfite during the analysis of NDF. The content of lignin was determined by dissolving cellulose with sulphuric acid [16]. The hemicellulose (HCL) content was calculated by the difference between NDF and ADF, and the cellulose (CL) content was calculated as the difference between ADF and lignin, which were expressed on a dry matter (DM) basis. The crude protein (CP) content was analyzed by the Kjeldahl method [17]. The water-soluble carbohydrate (WSC) content was determined using the method of Owens et al. [18]. Crude ash content was determined by complete combustion of the samples to a constant weight at 550 °C in a muffle furnace until a constant weight was recorded [19].

### 2.4. Structure

#### 2.4.1. Fourier Transform Infrared Spectroscopy

Infrared spectroscopy is widely studied in agricultural biomass materials. When the infrared light is irradiated on the sample, the molecules in the sample will absorb radiation of certain frequencies, causing vibration and rotation of the molecules, and the absorption spectrum is called the infrared spectrum. Each functional group has its own specific infrared spectral absorption region, so it is often used to characterize the functional groups of biomass.

The changes in functional groups of buckwheat straw before and after expansion treatment were conducted by an FTIR spectrometer (iS10, Nicolet Corporation, Madison, WI, USA). Buckwheat straw samples were dried at 105 °C for 4 h and stored in a desiccator before analysis. The sample spectra were obtained by scanning 32 times in the spectral range of 400–4000 cm^−1^, with a spectral resolution of 4 cm^−1^. The samples were analyzed by comparing the spectra for changes in chemical bonds.

#### 2.4.2. X-ray Diffraction

X-rays are scattered when they are irradiated on crystalline cellulose, and the scattered X-ray is strengthened at some specific angles, thus showing the characteristic diffraction phenomenon of crystalline cellulose. The crystallinity of cellulose can be obtained by X-ray atlas, which refers to the proportion of crystalline cellulose. Crystallinity is an important index affecting the conversion rate of cellulose.

The cellulose crystallinity index (CrI) of Control, EO, and ES was obtained by an X-ray diffractometer (Purkinje, XD3, Beijing, China) using Cu/Kα irradiation at 36 kV and 20 mA. All buckwheat straw samples were scanned from 5° to 40° with a step size of 0.02°. The CrI was calculated using the method of Segal et al. [20].

#### 2.4.3. Scanning Electron Microscopy

Changes in the surface morphology of buckwheat straw were visually examined using a scanning electron microscope (SEM). All dried samples were attached to the sample stage with conductive tape and then coated with a thin layer of gold with a vacuum coater. Finally, the samples were viewed using an SEM SU3500 (Hitachi, Tokyo, Japan) with an accelerating voltage of 15 kV.

### 2.5. Mechanical Properties

The mechanical properties directly affect the palatability, compression deformation, and even the flow properties of straw during digestion. The texture properties of straw can reveal changes in hardness, chewability, elasticity, and cohesiveness, and the rheological properties, including the stress relaxation and creep of straw, can reflect the resistance to internal structural deformation of straw under pressure and the deformation and flow properties under external forces.

#### 2.5.1. Dynamic Compression Test on the Texture Properties

Dynamic mechanical experiments were selected to compare their mechanical properties using a Texture Analyzer (TAXT2i, Stable Micro Systems, Surrey, South East England, UK). Texture profile analysis (TPA) tests were conducted in two-cycle compression mode with a P/50 cylindrical probe at 1 mm/s speed to 75% of the original height of the samples. The probe stayed for 5 s after the first depression, then started an upward stroke at a speed of 1 mm/s, and the second compression-decompression cycle was performed later. To more accurately evaluate the effect of expansion on the texture characteristics of buckwheat straw, the remaining undried samples were used for the tests. The raw material was wetted to a moisture content of 38% before the compression test to ensure that the moisture content was at the same level and avoid being affected by the large difference in moisture content. Similarly, in order to eliminate the influence of compression density, 10 g samples were weighed and put into a 150 mL beaker (diameter about 50 mm, height 70 mm) before the experiments, and the height of each sample was guaranteed to be 50 mm. The load cell was calibrated with a 1 kg weight with a 1 g least count. Each compression test for each sample was repeated five times. Calculated TPA parameters related to hardness, springiness index, cohesiveness, and chewiness from the output force-time curves [21].

#### 2.5.2. Static Compression Test on the Rheological Properties

Stress relaxation and creep tests were carried out with a P/50 probe at 1 mm/s test speed using a Texture Analyzer (TAXT2i, Stable Micro Systems, Surrey, South East England, UK), obtaining the curves of force-time and deformation-time through compression mode test, respectively [22]. The resulting force-time curves were developed for the calculation of compressive stress and relaxation elasticity, and deformation-time curves were recorded as the creep deformation.

### 2.6. In Vitro Rumen Digestibility

In vitro rumen digestibility was performed according to Tilley and Terry [23]. Fresh rumen fluid collected in the morning from three Angus bullocks fed with corn silage and a wheat straw-based diet was filtered through four layers of cheesecloth to remove large feed particles and mixed with the buffer solution in a 1:4 (*v*/*v*) ratio under continuous flushing with CO_2_. The samples of different expanding degrees were weighed (approximately 500 mg of each sample) into filter bags, then placed in a Daisy II Incubator and incubated at 39 °C for 48 h. The filter bags were retrieved after digestion and rinsed with water until the water was clear. Then, the rinsed filter bag was gently squeezed and dried at 65 °C for 48 h. Digestibility was calculated based on changes in DM, NDF, ADF, CL, and HCL contents.

The air-dried samples were weighed (220 mg each) into a 100 mL glass syringe. Each syringe received 30 mL of the buffered rumen fluid and was incubated at 39 °C for 96 h to obtain gas production. It was recorded manually at 0, 2, 4, 6, 8, 10, 12, 16, 20, 24, 30, 36, 42, 48, 56, 64, 72, 84, and 96 h of incubation. The kinetics of gas production were fitted using the NLIN procedure of SAS version 9.4 (SAS Institute, Cary, NC, USA), according to France et al. [24].

Model 1: Y = B (1 − e − c (t − L))(1)

Model 2: Y = B_1_ (1 − e − c_1_ t_1_) + B_2_ (1 − e − c_2_ t_2_) (2)

where Y (mL/g DM) is the volume of gas production at time t; B (mL/g DM) is the asymptotic gas production; B_1_ and B_2_ (mL/g DM) are the asymptotic gas production of the rapid or slow fermentation component; c (h^−1^) is the rate of gas production; c_1_ and c_2_ (h^−1^) are the rates of the rapid or slow fermentation component; and L (h) is the lag time prior to gas production.

### 2.7. Statistical Analysis

The statistical analysis was analyzed by one-way analysis of variance (ANOVA) followed by Tukey’s multiple range tests and declared when *p* < 0.05 using IBM SPSS 25 for Windows (IBM SPSS Inc., Chicago, IL, USA). FTIR spectra were baseline corrected and normalized using OMNIC 32 (Nicolet, New York, NY, USA). Graph processing was performed using Origin 2023 for Windows (Origin Lab Corporation, Northampton, MA, USA). Pearson analysis was used for correlation analysis.

## 3. Results

### 3.1. Changes in Chemical Components

Expansion led to a downward trend in lignocellulose components, while WSC and CP showed an upward trend (*p* < 0.05, Table 1). There was a significant difference in NDF content between EO and ES (*p* < 0.05), which was mainly caused by the decrease in lignin and hemicellulose content. The lignin content decreased in EO but was not significant, while it decreased significantly in ES (*p* < 0.05). Hemicellulose also had an obvious loss in both expansion processes (*p* < 0.05). The crude ash content did not change significantly.

### 3.2. Analysis in Structure

#### 3.2.1. FTIR Spectra

**Figure 1:**
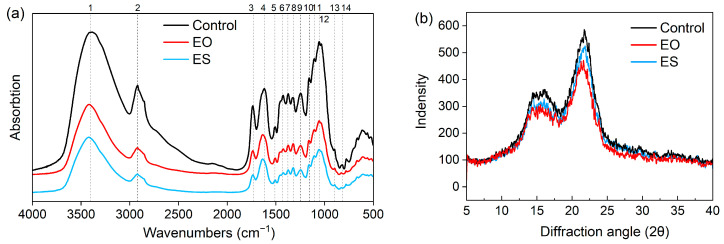
Structural changes in chemical bonding with FTIR (**a**) and crystallinity with XRD (**b**). Control, untreated; EO, once expansion; ES, secondary expansion.

Figure 1a indicates the variation of the absorption intensity of chemical bonds with different pretreatments, and Table S1 reveals the changes in the absorption peak position of chemical bonds. The absorption peaks near 1737 cm^−1^ and 898 cm^−1^ weakened by expansion. Compared to Control, the absorption peak at 1629 cm^−1^ and 3418 cm^−1^ was significantly blue-shifted. The absorption peaks at 1506 cm^−1^ and 1425 cm^−1^ related to the vibration of the benzene ring showed a decrease in EO and ES. The positions of 1319 cm^−1^ and 1245 cm^−1^, which represent the structural units of syringyl and guaiacyl, respectively, were shifted to different degrees. The vibration frequency decreased after pretreatment to 2920 cm^−1^.

#### 3.2.2. Diffraction Pattern

The XRD curves in Figure 1b displayed that the expanded samples had crystalline regions and amorphous regions of cellulose around 2θ = 22° and 2θ = 18°, respectively. It could be seen that the diffraction intensity of the Control, ES, and EO decreased successively. Using the Segal formula to calculate the crystallinity index of different treatments from the corresponding XRD patterns and the CrI of the Control, the EO, and the ES were 56.58%, 51.21%, and 52.79%, respectively.

### 3.3. Analysis of Mechanical Properties

#### 3.3.1. Texture Properties Based on Texture Profile Analysis

**Figure 2:**
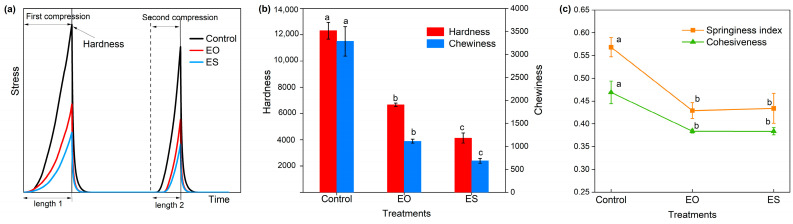
Compressive curves of TPA and calculation results of the texture properties of different treatments. (**a**) Two cyclic compression curves in texture analysis; (**b**) Hardness and chewiness calculated from compression curves; (**c**) Springiness index and cohesiveness calculated from compression curves. Control, untreated; EO, once expansion; ES, secondary expansion. Significant differences between treatments are indicated by different letters (*p* < 0.05).

Texture profile analysis (TPA) includes two compression cycles as shown in Figure 2a, and the hardness, chewiness, springiness index, and cohesiveness of materials were calculated according to the curves (Figure 2b,c). The results showed that hardness was reduced by about 50% in EO compared with the Control and decreased by about 66% in ES. Chewiness showed a similar trend to hardness. The highest springiness index and cohesiveness were obtained in Control, while they decreased by 25% and 18%, respectively, after expansion and had no significant difference in EO and ES.

#### 3.3.2. Rheological Properties Based on Stress Relaxation and Creep Tests

**Figure 3:**
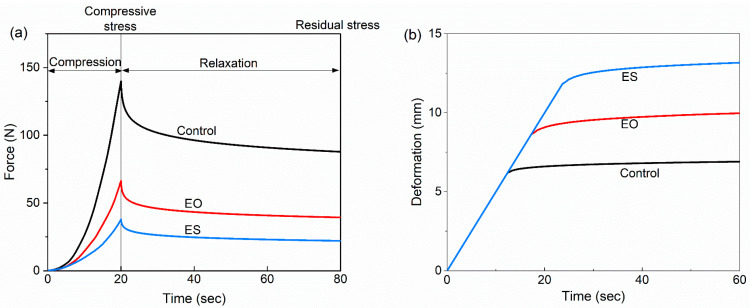
Compressive curves of stress relaxation and creep deformation curves of different treatments. (**a**) Representation of compressive stress and relaxed elasticity; (**b**) Representation of creep deformation. Control, untreated; EO, once expansion; ES, secondary expansion.

Figure 3 displayed the stress relaxation and creep curves, which showed the force and deformation of the sample over time, respectively. The compressive stress decreased by about 54% in EO and 73% in ES (*p* < 0.001, Table S2). The relaxation elasticity of the expanded samples decreased by about 5%, and there was no significant difference between the EO and ES. Under the same load condition, the creep deformation increased from about 6 mm in Control to 10 mm in EO and 13 mm in ES (*p* < 0.001).

### 3.4. In Vitro Rumen Digestibility

**Figure 4:**
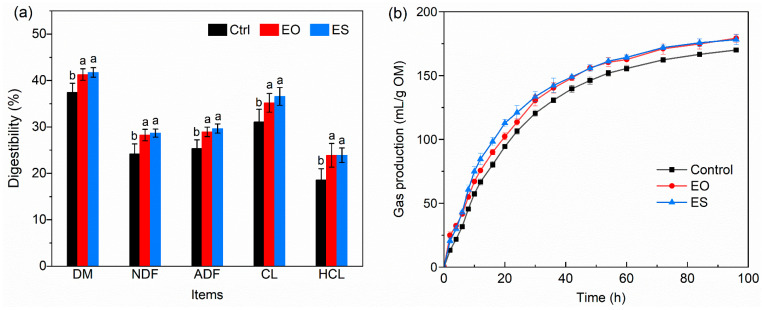
In vitro digestibility of the raw and treated buckwheat straw. (**a**) Degradation of each component after incubation for 48 h; (**b**) Gas production during incubation for 96 h. SEM represents standard error of the mean, n = 6. Significant differences between treatments are indicated by different letters (*p* < 0.05).

After 48 h incubation, expanded samples showed significantly higher digestibility of DM, NDF, ADF, CL, and HCL compared to the Control (Figure 4a, *p* < 0.05). The EO and ES increased the digestibility of DM, NDF, ADF, CL, and HCL by 3.8–5.3%, 4–5.1%, 3.7–5%, 4.6–6.0%, and 5.3%, respectively. Figure 4b showed that the Control had the lowest gas production, and there was a gas production delay period. There was no significant difference in gas production between EO and ES after 96 h of incubation. The results of gas production model fitting showed that model 1 was the best fit for Control, while model 2 was the best fit for EO and ES. The maximum theoretical gas production of EO and ES was composed of fast fermentation components and slow fermentation components, and the EO was slightly higher than the ES, whereas the gas production rates in ES were higher than those in EO (Table 2).

### 3.5. Changes in Morphological Composition

**Figure 5:**
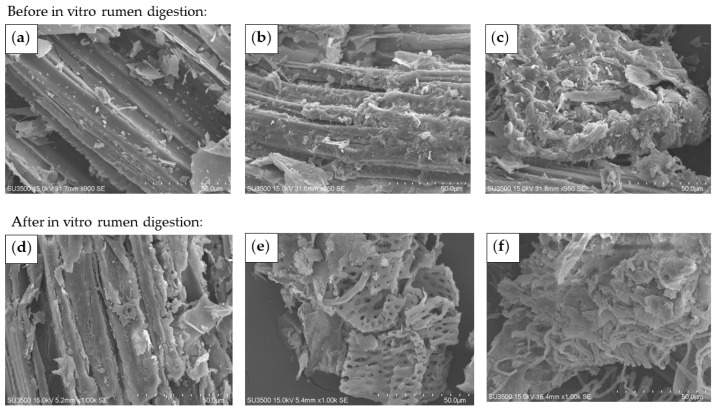
Scanning electron microscopy (SEM) of buckwheat straw before and after in vitro rumen digestion. (**a**–**c**) Untreated, once-expanded, and secondary-expanded buckwheat straw before digestion; (**d**–**f**) Untreated, once-expanded, and secondary-expanded buckwheat straw after digestion.

Figure 5a illustrates the smooth surface and ordered fibers of the Control. However, the exposed fibers of the EO were rough with deposits (Figure 5b), and the fiber structure of the ES was further destroyed and disordered, even displaying a honeycomb shape (Figure 5c). After in vitro rumen fermentation, cracks and holes could also be observed on the surface of the Control, but there was still an obvious lignified structure (Figure 5d). However, the fiber in the EO curled and the pore structure greatly increased (Figure 5e), and the samples of the ES had more deposits on the surface and had no visible complete fiber structure (Figure 5f).

### 3.6. Correlation of Mechanical Properties, Chemical Characteristics, and Gas Production Rate

**Figure 6:**
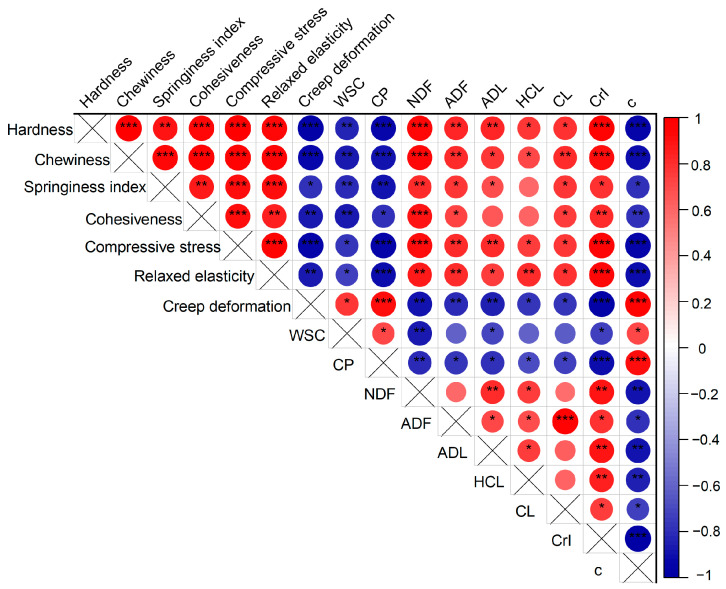
Correlation of mechanical properties, chemical characteristics, and gas production rate. Positive correlations were shown in red, and negative correlations were shown in blue. The dot color and size were proportional to the correlation coefficient, and Pearson’s correlation coefficient and the corresponding color were shown on the ruler on the right side. × means that correlations between the same items are ignored. * *p* ≤ 0.05, ** *p* ≤ 0.01, *** *p* ≤ 0.001.

The internal relationship between the change law of mechanical properties and chemical properties, as well as the efficiency of the gas production process during the expansion, is shown in Figure 6. Hardness, chewiness, springiness index, cohesiveness, compression stress, and relaxation elasticity all had a strong positive correlation with NDF content. Hardness and compression resistance were strongly correlated with lignin (*p* < 0.01), chewiness had a strong correlation with cellulose (*p* < 0.01), and relaxation elasticity was correlated with hemicellulose (*p* < 0.01). Hardness, chewiness, compression stress, and relaxation elasticity were significantly positively correlated with crystallinity (*p* < 0.001), while negatively correlated with gas production rate (*p* < 0.001). The deformation was negatively correlated with fiber composition and crystallinity while positively correlated with gas production rate (*p* < 0.001). There was a significant negative correlation between crystallinity and gas production rate (*p* < 0.001). The content of CP and WSC was negatively correlated with the mechanical properties of straw except for creep deformation, while positively correlated with the gas production rate.

## 4. Discussion

### 4.1. Changes in Chemical Components

The EO did not significantly contribute to reducing lignin content, but the ES significantly reduced the lignin content of buckwheat straw. Lignin has been reported to have a lower melting point of approximately 140 °C, and it becomes soft and sometimes melts when biomass is heated [25]. Since the extrusion process is shorter in time and the lignin structure is hard, the fast rotating speed of the screw and the rise in temperature during the EO might lead to the softening of the lignin and the destruction of the structure to a greater extent. As the structure of the straw had been preliminarily destroyed in the EO, more lignin was removed in the ES when the softened straw was placed in the high-temperature and high-pressure environment again. Hemicellulose content was degraded less in EO, which might be attributed to the shorter expanding time of the raw material, and it was discharged from the discharge port before being degraded in large quantities. However, hemicellulose was largely degraded in ES due to its amorphous state and low polymerization degree under high temperature and high pressure. Some studies have also shown that the thermally unstable acetyl division of hemicellulose can release acetic acid, which can catalyze the hydrolysis of lignocellulosic biomass [26]. The plant cell wall mainly consists of the intercellular layer, primary wall, and secondary wall. The main chemical components of the intercellular layer are lignin and pectin. The primary wall contains a lot of hemicellulose and lignin, and the secondary wall mainly consists of cellulose and hemicellulose. Through the analysis of the changes in lignocellulose components, it can be inferred that expansion mainly destroys the intercellular layer and primary wall structure. The increase in WSC content after expansion was possibly due to the fact that expansion broke the seal of the lignin, and partial hemicellulose or cellulose was hydrolyzed under acidic conditions and elevated temperatures to oligo- or monosaccharides [27]. However, WSC content did not increase significantly in ES compared to EO, probably because the acidic environment could result in the formation of some inhibitors. The principle of the Kjeldahl nitrogen determination method for detecting crude protein content is to measure the element content of nitrogen in straw and multiply it by 6.25 to obtain the crude protein content. Nitrogen is impossible to change during the expansion process, so the absolute content of crude protein is also impossible to change. However, due to the action of high temperature, thermal mechanical energy, and water steam, some soluble substances such as starch, fat, and sugar would be lost in the expansion process, resulting in an increase in the proportion of nitrogen elements.

### 4.2. Analysis in the Chemical Structure

FTIR spectra reflected modifications of the chemical bond structure of lignocellulose. The signal at 1629 cm^−1^ represents the stretching vibration of C=O in the conjugated carbonyl group. The peak position was blue-shifted in EO and ES, suggesting that expansion could destroy the structure of the conjugated carbonyl group and that lignin is easier to oxidize. The expanded samples showed a decrease in the aromatic skeleton vibrational intensity at 1507/1506 cm^−1^ and in the aromatic vibration intensity at 1425/1424 cm^−1^, indicating that aliphatic and aromatic groups in lignin were degraded. Moreover, the shift of characteristic peaks and changes in absorption strength of lignin structural units, including guaiacyl (1245/1246 cm^−1^), syringyl (1323~1319 cm^−1^), and *p*-hydroxyphenyl (831~828 cm^−1^), demonstrated the modification of lignin structure by expansion. It has been reported that the lignin extraction rate of expanded rice straw is high, and expansion is considered to be an effective method to improve the contribution of lignin separation [28]. In the present study, changes in chemical bond structure also demonstrated that although the EO had no significant effect on the degradation of lignin, it destroyed the tight connection between lignocellulose. The reduction of the absorption peak at 1737 cm^−1^ indicated that the expansion effectively broke the ester bond of lignin and hemicellulose, and the red-shifted of peak position in ES indicated that the damage degree was more serious, which was consistent with the decrease of hemicellulose. Declines of peaks intensities at 1374/1373 cm^−1^ represented C-H deformation of crystalline cellulose and hemicellulose, and 898 cm^−1^ represented C-O-C stretching of amorphous cellulose [29] suggested that both cellulose and hemicellulose structure were destroyed during expansion. The changes at 3418 cm^−1^ indicated that the degree of O-H association was reduced [30] and the hydrogen bond was broken during expansion. Chemical bonds determine the reaction rate of components since the different types and quantities of chemical bonds connected between molecular polymers determine the energy required [13]. Therefore, the destruction of chemical bonds and the reduction of the number of chemical bonds would promote the reaction rate of the digestive system.

The crystallinity index (CrI) value reflected the sample’s crystallinity and sensitivity to other components, including amorphous cellulose, hemicellulose, and lignin, which together contribute to the amorphous portion [31]. Buckwheat straw was extruded, sheared, and rubbed during expansion, then exploded instantly at the discharge port, resulting in a size reduction of lignocellulose as well as depolymerization [32,33]. With the secondary expansion, high-pressure steam further produced mechanical separation and tearing of fibers [34], destroying the polarity of cellulose molecules and the interaction between molecular chains. However, the ES resulted in a decrease in amorphous structures such as lignin and hemicellulose, which increased the proportion of crystalline regions, leading to a slightly higher crystallinity compared to the EO. The partial expansion of amorphous regions might improve the nutritional availability of pretreated feeds since this loosely agglomerate structure enables water and enzymes to access the hydrolytic reaction [35].

### 4.3. Analysis of Mechanical Properties

The mechanical properties of lignocellulosic materials are important factors affecting the conversion efficiency, which affects energy consumption and material transfer efficiency of the digestive system. TPA mainly reflects the hardness, elasticity, chewability, and cohesiveness of the straw itself, and stress relaxation and creep deformation can reflect the rheological characteristics of the system after straw compression. Hardness is an indicator used to measure the soft and hard texture of samples, and chewiness is a description of solid samples used to simulate the energy expended when the sample is chewed to a state that can be swallowed. During the expansion process, the straw was blasted into 1–2 cm fragments under the dual action of high-temperature effect and mechanical effect, and the cell wall disintegrated [36], resulting in a decrease in the hardness of the straw. Compressive stress refers to the force required to resist the deformation of the internal structure of the biomass material, and the expanded samples required less force to be compressed. Creep deformation represents the deformation of the samples under the same load condition, reflecting the degree of difficulty of the sample being compressed. The expansion broke the ordered structure of cell wall, resulting in larger deformation under the same load. Springiness index indicates the recovery ability of deformation. Expanded straw had the lower springiness index, indicating that expanded biomass was not easy to rebound after compression, which is conductive to the silage compaction of biomass. Cohesiveness refers to the ratio of compression work between the second cycle and the first cycle and reflects the inner structure force of the sample. The cohesiveness of expanded buckwheat straw decreased. The in-depth study of physical properties provides a theoretical basis for the improvement of the palatability and compression performance of silage.

### 4.4. Analysis of In Vitro Rumen Digestibility

It was found that the expansion significantly improved the utilization rate of nutrients through the analysis of in vitro digestibility and gas production. The findings were also supported by chemical and physical property modifications. The reduction of the mechanical properties of the expanded straw increased the gas production rate. The disruption of the lignocellulose structure and separation of fibers increased the contact area of enzymes. However, the further decrease in hardness and compressive stress in ES did not lead to further improvement in digestibility and gas production. It was speculated that the high deposition density of lignin droplets covered the fiber surface and blocked part of the pore structure [37], resulting in limited space for the attachment of microorganisms. SEM images seemed to demonstrate the opinion, which was due to the gradual separation of tightly bound cellulose microfibers and the interaction of thermal expansion and water, eventually causing lignin to be melted, solubilized, and recondensed on the surface [38]. Interestingly, the gas production rate was further increased along with the decrease in hardness. It was inferred that the weakening of the mechanical properties of the expanded straw reduced the energy consumption required to overcome the internal structural force during digestion in rumen, thus promoting the improvement of conversion efficiency. Although some similar pretreatment methods could achieve higher digestion rates, such as steam explosion, which increased the degradation rates of NDF, ADF, CL, and HC by 10.53%, 7.84%, 5.79%, and 13.04%, respectively, severe treatment conditions sacrificed more than 50% of hemicellulose and other nutrients and even produced by-product inhibitors such as furfural and hydroxymethylfurfural [39]. There is also a study indicating that the loss of a large amount of fiber components during steam explosion treatment of wheat, rice, and corn straw results in no significant change in the in vitro gas production after 96 h compared to the raw materials, only accelerating the gas production rate [40]. Therefore, expansion can improve the utilization value of buckwheat straw without causing a lot of nutrient loss.

### 4.5. Correlation Analysis of Mechanical and Chemical Characteristics and Gas Production Efficiency

Different chemical components cause different mechanical properties owing to different contents and unique chemical bonds. It proved that hardness, chewiness, compressive stress, and deformation were strongly correlated with lignin and cellulose, indicating lignin and cellulose play the main role in hardness. Some studies have shown that reductions in the lignin content caused the stem mechanical strength to decrease, suggesting that lignin is closely tied to cell wall mechanical strength [41,42]. Cellulose, a glucose homopolymer composed of β-(1,4)-glucan chains, is the main component of the cell wall and exhibits strong rigidity due to intermolecular and intramolecular hydrogen bonding [43]. The degradation of lignin and cellulose caused by expansion led to a reduction in mechanical strength. Relaxation elasticity reflects the energy required by the sample to resist relaxation deformation when being compressed, which is positively correlated with hemicellulose. This was not only related to the hydrophilicity of hemicellulose but also to its role as a coupling agent between matrix and hard cellulose fibrils due to the spatial organization and structural diversity of hemicellulose [44]. The above analysis suggested that structural carbohydrates play important roles in the mechanical strength of straw. The decline in the hardness and relaxation elasticity of expanded straw improves the palatability and chewiness. Besides, these findings are beneficial to compression during silage, and the resilience of the feed is weakened, which reduces the infiltration of air and is beneficial to improving the aerobic stability of the feed.

Notably, crystallinity is a key factor affecting the digestibility and resistance of biomass. The crystallinity decreased, accompanied by a decrease in the amount of crystalline cellulose and degree of densification, thereby reducing the work to overcome the internal structural force of straw. Besides, the moisture content of the straw increased from 5% to about 38% after expansion. Some studies have shown that moisture can enter the cell wall and interact with the macromolecules by hydrogen bond and van der Waals force [30,45], changing the internal structure force of plant biomass. The swelling effect of cellulose can alter the mechanical properties of plant biomass [13].

Changes in physicochemical properties would lead to different chemical potential energy and reaction activation energy in the reaction process, resulting in different reaction rates. Mechanical properties and CrI have a stronger correlation with gas production rate. In vitro digestion process mainly relies on enzymes secreted by rumen microorganisms for enzymatic hydrolysis. Liu and Chen [13] proposed that the high cohesiveness of the enzymatic hydrolysis system affected the efficiency of enzymatic hydrolysis. This was due to the incompatibility between liquid and solid, which needs to overcome the resistance and surface tension between interfaces. The highly cohesive system would increase energy consumption during digestion. This study proved that the gas production rate increased with the decrease in cohesiveness. The decrease in cell wall stiffness caused by expansion supported by physical properties led to a decrease in internal structural forces. It could reduce the energy consumption required to overcome the internal structural forces during digestion, thus promoting gas production efficiency. The decrease in cellulose crystallinity was also conducive to in vitro rumen fermentation. The decrease in lignin content and crystallinity is accompanied by an increase in grain dislocation, which promotes the accessibility of catalysts to reach reactive sites in cellulose and thus improves digestibility [46,47]. In addition, the denatured protein was more easily hydrolyzed and better absorbed, which was consistent with the very significant positive correlation between crude protein and gas production rate. The results of this study showed that the modification of the physiochemical properties of straw by expansion had a favorable effect on digestibility. Given expanded straw utilization value enhancement, in the future we will further analyze expansion pretreatment in feed for the application of microbial metabolism and function [48].

## 5. Conclusions

Expansion effectively improved the physicochemical properties of buckwheat straw to enhance its utilization as a feed source. Expansion resulted in a 4–7% decrease in NDF content, partial hydrolysis of hemicellulose, and redistribution of lignin. Compared with chemical composition, the improvement of mechanical properties more reflected the bioavailability of straw as fermented feed. The significantly reduced hardness and elasticity of the expanded straw improved the chewiness and palatability, which were related to the lower CrI and chemical bond energy. The weakening of mechanical properties reduced the energy required to overcome internal structural forces during digestion, thereby increasing in vitro gas production efficiency. The secondary expansion might have only a small component modification effect on the damaged lignocellulose structure and could not significantly increase digestibility, but it could cause a significant decrease in hardness. Expansion pretreatment significantly improved the utilization value of straw and solved the problems of high lignification degree, poor palatability, and difficult compression of the dry straw. This study lays a theoretical foundation for research on expansion applied in silage fermentation or compression transportation so as to further improve the nutritional value of straw as ruminant feed.

## Figures and Tables

**Table 1:** Chemical compositions of buckwheat straw in different treatments.

Items (g/kg DM)	Control	EO	ES	SEM ^1^	*p*-Value ^2^
Neutral detergent fiber (NDF)	735.8 a	705.8 b	688.6 c	7.01	<0.001
Acidity detergent fiber (ADF)	584.7 a	554.9 b	542.9 b	6.93	0.004
Lignin	84.9 a	81.9 a	76.5 b	1.39	0.007
Cellulose (CL)	498.0	472.3	463.7	6.87	0.086
Hemicellulose (HCL)	154.1 a	151.5 ab	145.7 b	1.36	0.017
Water-soluble carbohydrate (WSC)	16.7 b	21.5 a	21.6 a	0.94	0.017
Crude protein (CP)	32.1 c	39.5 b	43.7 a	1.71	<0.001
Crude ash	92.9	96.8	95.5	1.31	0.541

Control, untreated; EO, once expansion; ES, secondary expansion. ^1^ SEM represents the standard error of the mean, n = 3. ^2^ Significant differences in each row are indicated by different letters (*p* < 0.05).

**Table 2:** Kinetic parameters of in vitro gas production models.

Treatment ^1^	Y ^2^ (mL/g DM)	B ^3^ (mL/g DM)		c ^4^ (h^−1^)		L ^5^ (h)
		B_1_	B_2_	c_1_	c_2_	
Control	170.1	172.64	172.64	0.0398	0.0398	0.2038
EO	180.1	153.65	78.96	0.0523	0.0044	/
ES	178.2	123.54	75.67	0.0697	0.0139	/

^1^ Control, untreated; EO, once expansion; ES, secondary expansion. ^2^ Y, gas production at time t. ^3^ B, theoretical gas production; B_1_ and B_2_ represent theoretical maximum gas production of the rapid and slow fermentation component. ^4^ c, gas production rate; c_1_ and c_2_ represent gas production rates of the rapid and slow fermentation component. ^5^ L, lag phase.

## Data Availability

All the tables and figures used to support the findings of the current study are included in the article.

## Supplementary Materials

The following supporting information can be downloaded at: https://www.mdpi.com/article/10.3390/ani14010029/s1, Table S1: Signal assignments and relative intensities in FTIR spectra; Table S2: Changes in rheological properties.
