# Supplementary material for: Expansion Improved the Physical and Chemical Properties and In Vitro Rumen Digestibility of Buckwheat Straw
Source: Animals (Basel). 2023 Dec 21;14(1):29. doi: 10.3390/ani14010029 (PMC10777991; doi:10.3390/ani14010029)
Supplement: Supplementary file 1 [file animals-14-00029-s001.zip › animals-2750550-SI.pdf]

Tables S1. Signal assignments and relative intensities in FTIR spectra.

Peak	Attribution and description of FTIR absorption	Wavenumber (cm^{-1})		
		Control	EO	ES
1	Hydrogen bonded stretching absorption	3418	3418	3422
2	Aliphatic CHx stretching vibration	2920	2919	2918
3	Unconjugated C=O in hemicellulose	1737	1737	1736
4	Aromatic vibration	1629	1636	1636
5	Aromatic skeletal vibration	1506	1507	1507
6	Aromatic vibration	1425	1425	1424
7	C-H deformation	1374	1374	1373
8	C-O vibration of syringyl	1319	1323	1323
9	C-O vibration of guaiacyl	1245	1246	1246
10	C-O-C asymmetric stretch	1160	1161	1160
11	Aromatic C-H deformation in syringyl	1107	1108	1108
12	C-O stretch	1057	1056	1056
13	C-O-C stretching of amorphous cellulose	898	897	897
14	Aromatic C-H deformation out of plane	831	829	828

Table S2. Changes in rheological properties.

Items	Control	EO	ES	SEM[1]	*P*-value[2]
Compressive stress (N)	137.40a	63.81b	37.70c	10.14	<0.001
Relaxed elasticity (%)	62.55a	59.25b	57.84b	0.73	0.001
Creep deformation (mm)	6.76c	10.52b	13.12a	0.94	<0.001

Control, untreated; EO, once expansion; ES, secondary expansion. [1]SEM represents standard error of mean, n = 3. [2]Significant differences in each row are indicated by different superscripts ($P < 0.05$).
